# Oxygen Consumption Can Regulate the Growth of Tumors, a New Perspective on the Warburg Effect

**DOI:** 10.1371/journal.pone.0007033

**Published:** 2009-09-15

**Authors:** Yijun Chen, Rob Cairns, Ioanna Papandreou, Albert Koong, Nicholas C. Denko

**Affiliations:** 1 Division of Radiation and Cancer Biology, Department of Radiation Oncology, Stanford University School of Medicine, Stanford, California, United States of America; 2 Department of Surgery, Stanford University School of Medicine, Stanford, California, United States of America; Roswell Park Cancer Institute, United States of America

## Abstract

**Background:**

The unique metabolism of tumors was described many years ago by Otto Warburg, who identified tumor cells with increased glycolysis and decreased mitochondrial activity. However, “aerobic glycolysis” generates fewer ATP per glucose molecule than mitochondrial oxidative phosphorylation, so in terms of energy production, it is unclear how increasing a less efficient process provides tumors with a growth advantage.

**Methods/Findings:**

We carried out a screen for loss of genetic elements in pancreatic tumor cells that accelerated their growth as tumors, and identified mitochondrial ribosomal protein L28 (MRPL28). Knockdown of MRPL28 in these cells decreased mitochondrial activity, and increased glycolysis, but paradoxically, decreased cellular growth in vitro. Following Warburg's observations, this mutation causes decreased mitochondrial function, compensatory increase in glycolysis and accelerated growth in vivo. Likewise, knockdown of either mitochondrial ribosomal protein L12 (MRPL12) or cytochrome oxidase had a similar effect. Conversely, expression of the mitochondrial uncoupling protein 1 (UCP1) increased oxygen consumption and decreased tumor growth. Finally, treatment of tumor bearing animals with dichloroacetate (DCA) increased pyruvate consumption in the mitochondria, increased total oxygen consumption, increased tumor hypoxia and slowed tumor growth.

**Conclusions:**

We interpret these findings to show that non-oncogenic genetic changes that alter mitochondrial metabolism can regulate tumor growth through modulation of the consumption of oxygen, which appears to be a rate limiting substrate for tumor proliferation.

## Introduction

Otto Warburg won the Nobel Prize for physiology or medicine in 1931, and we are still working to understand the significance of his discoveries. His work on oxidation and reduction and energy production is essential for our current understanding of intermediate metabolism. Using his newly developed techniques, he characterized the energy production within solid tumors and compared it to that in normal tissue [1]. He found that normal tissues used mitochondrial oxidation to account for 90% of ATP production with glycolysis accounting for 10%. However, tumors used less of the highly efficient oxidative phosphorylation, producing 50% of the ATP from oxidation and 50% from glycolysis. This shift was thought to occur even though there was sufficient oxygen to support mitochondrial function and is called “aerobic glycolysis”[1]. Although Warburg incorrectly attributed the cancer phenotype to this metabolic shift, it is difficult to explain the growth advantage that tumor cells gain by increasing glycolysis at the expense of oxidative phosphorylation [2]. Glycolysis produces only two ATP per glucose molecule, along with 2 NADH and 2 molecules of pyruvate. Without mitochondria to consume the pyruvate, it is converted to lactate and released into the extracellular space. The conversion of pyruvate to lactate maintains the redox balance within the cell by regenerating NAD+, but wastes this energy rich molecule.

Genetic analysis of tumors has since identified oncogenic changes that have been shown to contribute to the altered tumor metabolism. Activation of oncogenes such as AKT [3] and myc [4], [5] have been shown to increase glycolysis and glutaminolysis, while loss of tumor suppressors TP53 [6] or VHL [7] can also shift energy production away from the mitochondria to a more glycolytic equilibrium. VHL has been especially informative due to its ability to directly regulate the hypoxia-inducible transcription factor 1α (HIF1α) [8]. HIF1α is proteolytically regulated by pVHL primarily by ambient oxygen concentrations. Low levels of oxygen (or hypoxia) cause the protein to be stabilized and become active in transcribing genes thought to allow the cell or tissue to adapt to the hypoxia. Recent work has shown that not only does HIF1 actively increase glycolysis [9] but it decreases mitochondrial function [10], [11], [12] in order to metabolically reduce the demand of the tissue for oxygen.

In addition to the oncogenic and epigenetic changes within the tumor that can alter mitochondrial function, tumor mutations have been found in mitochondrial DNA, and in nuclearly encoded mitochondrial enzymes. These mutations have been identified in tumors such as head and neck [13], prostate [14], ovary [15], and liver [16]. However, it is difficult to determine the role of these mutations during tumorigenesis, partly because mitochondrial mutations accumulate naturally during aging [17]. Phenotypic analysis of cells harboring these mutations showed increased growth rates in vivo [13], [14], and in vitro as well [13]. Both of these reports attributed the growth differences to a decrease in mitochonrially produced reactive oxygen species (ROS) when cells were grown in normoxia. Neither group investigated their cells in hypoxia or other microenvironmental conditions found in the tumor. One suggestion that the tumor microenvironment may be important in understanding the biology of these mutations comes from Mithani et al who showed that these mutations are late events during tumor formation [18]. We interpret this late accumulation of mitochondrial mutations to mean that they provide a growth advantage at the stage of a macroscopic tumor, where microenvironmental stresses exist that can in turn regulate cellular growth.

In addition to mitochondrial DNA mutations, there have recently been tumor mutations identified in the enzymes of the TCA cycle. Through genetic studies, fumarate hydrolase and succinate dehydrogenase have been shown to be tumor suppressors [19], [20]. The mechanism by which they can suppress tumorgenicity has yet to be fully determined, but it has been shown that these mutations cause a buildup of TCA intermediates that can lead to stabilization of the HIF1 transcription factor [21]. This condition, termed pseudohypoxia, can increase aggressiveness of tumors through the induction of HIF1 target genes such as VEGF or lysl oxidase. Recently, an unbiased tumor sequencing project found another related enzyme, isocitrate dehydrogenase, mutated at high frequency in glioblastomas [22]. Although this is the cytoplasmic form of the enzyme, it is closely related to the mitochondrial form, and the physiologic significance of this mutation is not yet determined.

Poorly formed tumor blood vessels cause a bottleneck that limits oxygen supply to the growing tumor [23], [24]. Oxygen consumption within the tumor causes an imbalance with delivery resulting in hypoxia and its sequella [25]. Hypoxia has been shown to have many effects on tumor cells, the severity of the response is dependent on the level of oxygen deprivation [26], [27]. With respect to tumor growth characteristics, moderate hypoxia causes a slowing of tumor cell proliferation, while severe hypoxia causes outright cell death [27], [28]. These effects can be seen in vivo as well as modeled in vitro. Reducing hypoxia by decreasing oxygen consumption may be the most effective way to bring supply and demand back in balance and increase tumor growth [25]. Mitochondrial function is responsible for the majority of oxygen consumption within the cell, accounting for 70–90% of total oxygen consumption. Reducing mitochondrial function can therefore have a major effect upon total oxygen utilization. However, there are only two ways that a cell can generate energy, so a reduction in mitochondrial oxygen consumption must result in a compensatory increase in glycolysis.

In this work, we describe a novel unbiased screen for genetic loss that increased tumor cell growth in vivo. The most dramatic target identified was in mitochondrial ribosomal protein L28, and its knockdown resulted in a slower growth in vitro, but paradoxically, an accelerated growth in vivo. One of the effects of the knockdown is to change the cellular metabolism to more closely match the Warburg observations. Some of the differences in growth can be modeled by altering oxygen availability in vitro. However, these findings emphasize how the Warburg effect must be studied in the context of the tumor microenvironment where nutrient supply is limited by blood supply, unlike the growth conditions in vitro.

## Results

An unbiased ShRNA screen identified genes whose knockdown increased cell growth in vivo. Miapaca2 pancreatic cancer cells were infected with a lentivirus library containing ShRNA hairpins targeting 8600 open reading frames with 5 fold redundancy [29]. The infected cells were selected for puromycin resistance and then either injected into the flank of a nude mouse, or placed in a dish for growth in an incubator. After one week and four weeks samples were harvested and the enrichment of each ShRNA determined. The genes that showed the greatest increase in the in vivo samples when compared to the in vitro samples are listed in figure S1. At the top of the list is the mitochondrial ribosomal protein L28 (MRPL28). This is a nuclearly encoded protein that is required for the correct function of the mitochondrial ribosome, and synthesis of the mitochondrially encoded proteins [30].

**Figure 1:**
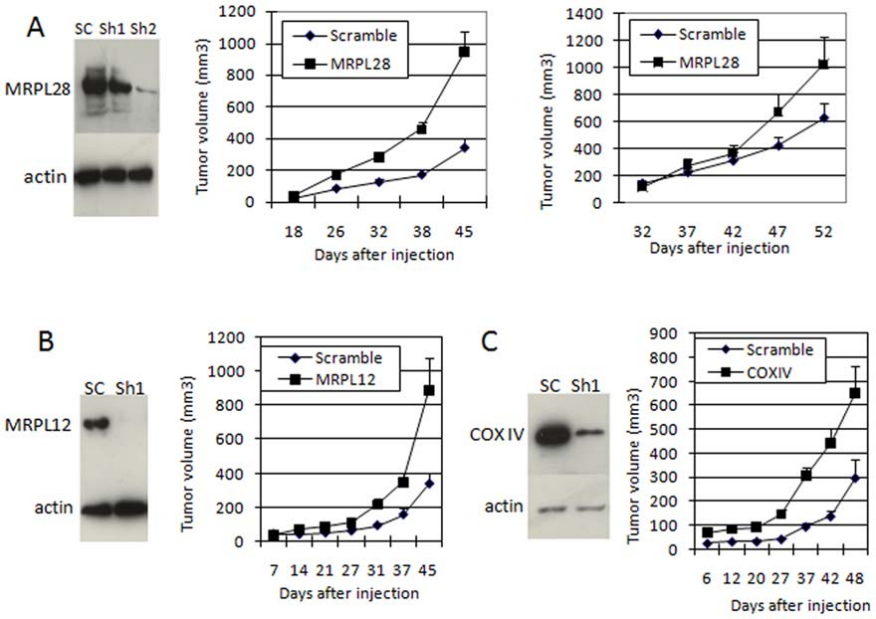
Knockdown of MRPL28 in pancreatic tumor cells increases tumor formation. Figure 1A) Left panel is a western blot showing knockdown of MRPL28 by ShRNA constructs 1 and 2 from figure S1 after infection of SU86cells. Middle panel shows tumor growth of SU86 cells infected with scrambled ShRNA or ShRNA2. Right panel shows growth of Miapaca2 cells after the infection with the indicated constructs. Figure 1B) Shows Western blot and increased tumor growth after knockdown of MRPL12 in SU86 tumor cells. Figure 1C) Shows western blot and increased tumor growth after knockdown of cytochrome oxidase subunit 4 in Su86 tumor cells.

To confirm that the knockdown of this protein would confer a growth advantage to cells grown as a tumor, two retroviral plasmids were constructed that expressed either hairpins 1 or 2 from table 1. Virus was made and used to infect both SU86 and Miapaca2 pancreatic cancer cells. Knockdown was determined by Western blot and found to be approximately 50% for construct 1 and >90% for construct 2 when compared to cells infected with a scrambled sequence hairpin (figure 1a). These cells were then injected into the flank of a nude mouse, and the rate of tumor growth determined by measuring the volume with calipers. Knockdown of MRPL28 in either of these cell lines resulted in approximately a 2–3 fold increase in tumor growth. Because MRPL28 is necessary for mitochondrial function, we tested the knockdown of other proteins essential to mitochondrial function to determine if this would phenocopy the MRPL28 knockdown. We found that knockdown of either mitochondrial ribosomal protein L12 or cytochrome oxidase subunit 4 resulted in a similar increase in tumor growth in vivo (figure 1b and 1c). We also found that expression of a truncated MRPL28 could increase tumor growth as well. (figure S2) This fragment appears to have dominant interfering activity in our growth and functional assays (figure S2).

**Figure 2:**
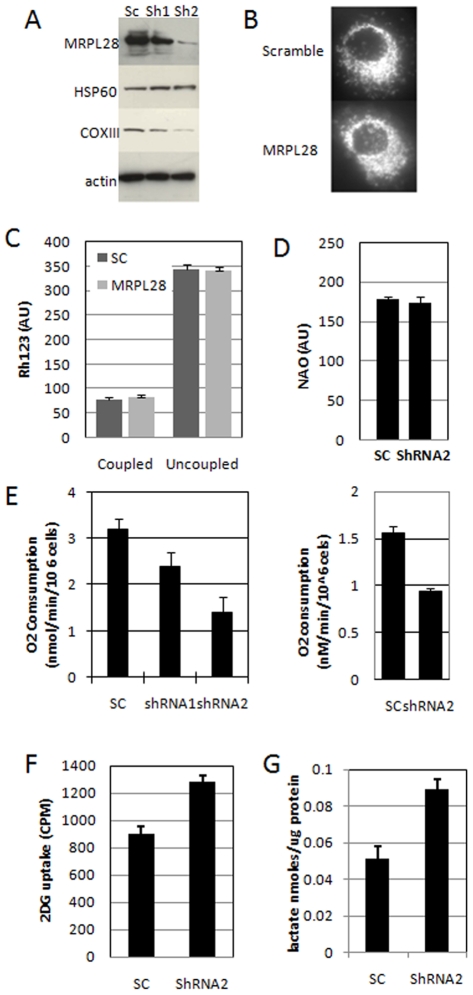
Knockdown of MRPL28 results in a dose-dependent decrease in mitochondrial function. Figure 2A) Western blot of the extracts from figure 1 probed for mitochondrially encoded COX111 and nuclearly encoded mitochondrial HSP60. Figure 2B) Micrograph of control and MRPL28 knockdown SU86 cells stained with anti-cytochrome C showing no significant alteration in mitochondrial morphology. Figure 2C) Determination of mitochondrial membrane potential by staining with Rhodamine123 in control and MRPL28 knockdown Miapaca2 cells showing no difference in either basal or uncoupled conditions (addition of 1 ug/ml CCCP). Figure 2D) Staining of total mitochondrial mass with nonyl acradine orange (NAO) in control and MRPL28 knockdown Miapaca2 tumor cells showing no difference. Panel 2E) Determination of reduced mitochondrial oxygen consumption in MRPL28 knockdown SU86 (left panel) and Miapaca2 cells (right panel). Panel 2F) Shows increased ^3^H-labelled 2-deoxyglucose uptake in the MRPL28 knockdown Miapaca2 tumor cells compared to the scrambled ShRNA control. Panel 2G) Shows increased lactate production in the media after 16 hours of growth of MRPL28 knockdown Miapaca2 cells when compared to scrambled ShRNA control cells.

To determine the role of MRPL28 knockdown on mitochondrial function, we analyzed the knockdown cells in vitro. We first used the protein extracts from figure 1 above, and probed for the expression of a mitochondrial protein that was encoded in the mitochondrial genome (COXlll), and one that was encoded in the nuclear genome (HSP60). We found that knockdown of MRPL28 resulted in a commensurate reduction in the mitochondrial protein, with little effect on the nuclear-encoded protein (figure 2A), suggesting that the mitochondrial ribosome was compromised. We examined the knockdown cells for any gross abnormalities in mitochondrial structure by immunostaining with anti cytochrome C antibody. Mitochondrial structure and quantity appeared normal by this assay (figure 2B). We also examined biochemical parameters of the mitochondria using the functional stain Rhodamine 123 (Rh123), and the mitochondrial stain Nonyl acradine orange. Figure 2C shows that the membrane potential (Rh123 intensity) and the total mitochondrial mass (NAO intensity) were not changed by the knockdown of MRPL28.

However, when we examined the metabolic parameters of these cells we found a significant effect of the knockdown. Figure 2E shows that mitochondrial oxygen consumption is significantly reduced in the knockdown of MRPL28 in both Su86 and Miapaca2 cells. We found that the greater the reduction in MRPL28 expression, the greater the reduction in the mitochondrial oxygen consumption (figure 2E). Reduced mitochondrial function suggests that these cells must have a compensatory increase in glycolytic energy production. Analysis of the knockdown cells for glucose uptake with radiolabelled glucose showed an increase relative to the control cells (figure 2F). Much of the increased glucose uptake appears to be used in glycolysis, as these cells also show an increase in lactate production as well. (Figure 2G)

**Figure 3:**
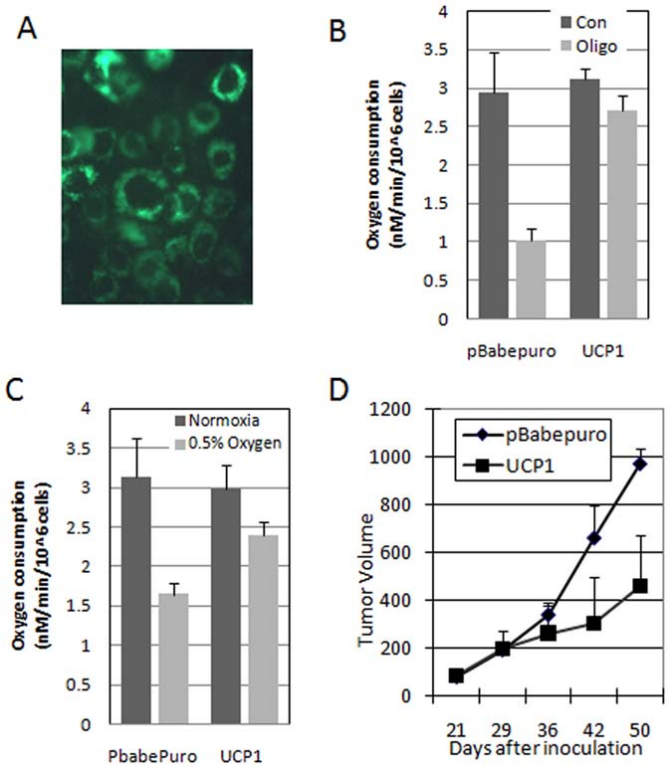
Increased mitochondrial oxygen consumption decreases tumor growth. Figure 3A) Immunodetection of stably transfected uncoupling protein 1 (UCP1) in Miapaca 2 cells. Note nearly 100% transduction. Figure 3B) Uncoupling of mitochondria by expression of UCP1. Mitochondrial oxygen consumption at baseline and in the presence of 1 ug/ml oligomycin to block the F1F0ATPAse. Note continued oxygen consumption in the uncoupled cells. Figure 3C) Increased oxygen consumption in UCP1 expressing Miapaca 2 cells exposed to hypoxia. Note similar oxygen consumption in normoxia. Figure 3D) Slower tumor growth in Miapaca2 cells expressing the UCP1 gene and presumably due to increased oxygen consumption.

In order to test the hypothesis that altering oxygen consumption can alter tumor growth, we generated cells with increased consumption to test if they showed slower tumor growth. To accomplish this, we overexpressed the mitochondrial uncoupling protein 1 (UCP1), which can dissipate the proton gradient across the inner mitochondrial membrane without generating ATP. The result is increased oxygen consumption without increased ATP generation. Figure 3A shows that over 90% of the cells express the introduced UCP1 protein by immune-fluorescence. These cells showed a significant level of uncoupling when mitochondrial function was tested in the presence of oligomycin (figure 3B). Oligomycin inhibits the F1F0 ATPase and blocks oxygen consumption in coupled mitochondria. Addition of oligomycin had little effect on UCP1 overexpressing cells, while it decreased oxygen consumption in control cells (figure 3B). However, UCP1 expression had little effect on baseline oxygen consumption (figure 3B and 3C). Because it is known that hypoxia can reduce mitochondrial function, we tested these cells after treatment with hypoxia, and found that in this environment that UCP1 expression resulted in significantly increased oxygen consumption when compared to control cells (Figure 3C). After determining that these cells showed increased oxygen consumption, we tested them for tumor growth, and found that as predicted, they did have decreased growth in vivo. (Figure 3D).

**Figure 4:**
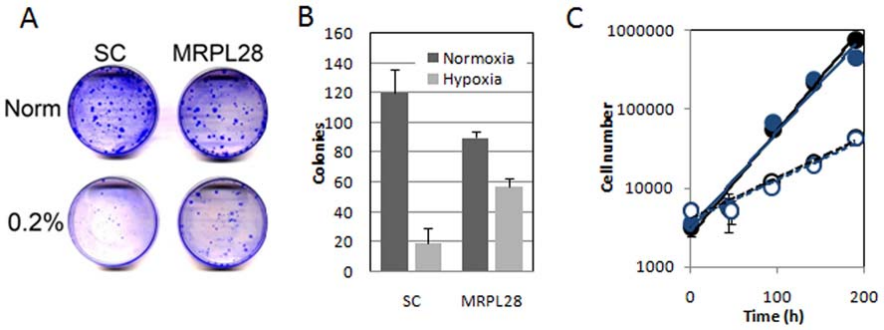
Knockdown of MRPL28 results in slower growth of tumor cells in vitro. Figure 4A) Xrystal violet stained colony formation of control scrambled ShRNA and MRPL28 knockdown Miapaca2 cells grown in normoxia and in o.2% oxygen. Figure 4B) Quantitation of plating efficiency shown in figure 4A. Note fewer colonies in normoxia in the knockdown cells, and less of a decrease in moderately severe hypoxic conditions. Figure 4C) Addition of the oxygen radical scavenger NAC does not rescue the growth of the normoxic MRPL28 Miapaca2 knockdown tumor cells. Growth of scrambled control ShRNA cells (closed symbols) and MRPL28 knockdown cells (open symbols). Cell number is reported for growth in control media (dark symbols), or media containing 10 mM NAC (light symbols).

Working with these cells in the lab, it appeared that the MRPL28 knockdown (and the MRPL12 and COX1V knockdown also) cells grew more slowly in vitro. We therefore tested the growth of MRPL28 knockdown cells for colony formation in normoxia, and hypoxia, a condition that would be prominent in vivo, and capable of regulating growth. We found that moderately severe hypoxia of 0.2% could greatly inhibit the growth of the control cells infected with the scrambled sequence hairpins. However, while the MRPL28 cells grew more slowly in normoxia, they were not inhibited as the parental cells were in hypoxia (figures 4A and 4B). This slow growth of the knockdown cells in 21% oxygen could be due to the accumulation of toxic oxygen radical byproducts that are reduced in hypoxia. We tested this hypothesis by measuring the growth rates of these cells in normoxia in media that had been supplemented with the scavenger N-acetyl cysteine. However, protection against ROS with this drug had no effect on the normoxic growth of the MRPL28 knockdown cells. (figure 4C).

**Figure 5:**
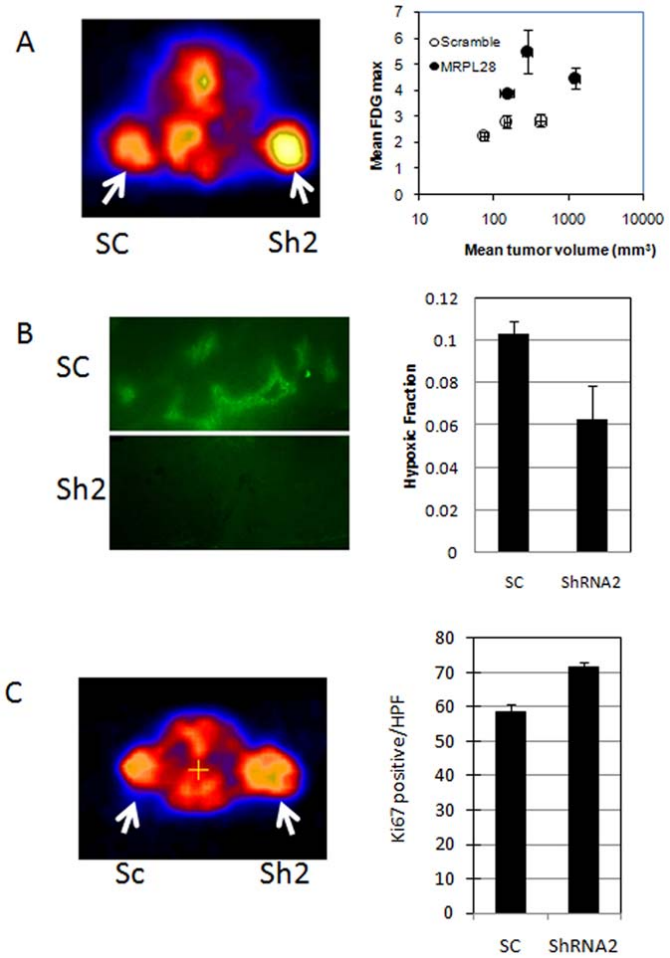
Metabolic changes cause accelerated growth the MRPL28 knockdown tumors. Figure 5A Glucose uptake by microPET determination of 18F labeled deoxyglucose. Animals bearing one control scrambled ShRNA tumor and one MRPL28 knockdown SU86 tumor were injected with 200 uCi FDG; and imaged one hour later. Left panel shows heat map with MRPL28 tumors with increased uptake. Right panel shows quantitation of FDG uptake is increased independent of tumor size. Figure 5B) Size matched control Scrambled ShRNA SU86 and MRPL28 knockdown tumor bearing animals were injected with pimonidozole and 3 hours later sacrificed. Tumors were sectioned and stained for pimonidozole binding. Representative sections are shown in left panel and quantitation of hypoxic fraction shown in right panel. Figure 5C) Tumor proliferation in vivo was determined by 18F labeled thymidine for cellular proliferation. Animals bearing one control scrambled ShRNA and one MRPL28 knockdown SU86 tumor were injected with 200 uCi of 18F-thymidine and two hours later imaged. The PET signal was marginally increased, so tumor cell proliferation was confirmed by Ki67 staining of sections taken from the tumors. Right panel reports Ki67 positive cells per high powered field.

These knockdown cells therefore seem to have a metabolism that functions more efficiently in vivo while sacrificing efficient growth in vitro. We therefore analyzed these metabolic and growth parameters in the tumors grown from cells with control and MRPL28 knockdown hairpins. Using the radiotracer 18F labeled 2-deoxyglucose, we measured glucose uptake in vivo. Even accounting for the larger size of the tumors, we found that the knockdown tumors had a 1.5–2 fold increase in glucose uptake (figure 5A). We also tested fresh tumor explants for oxygen consumption differences, and found that the knockdown tumors had significantly lower oxygen consumption per mg of tumor than the control tumors (figure S3). Because we had determined that the rate of oxygen consumption can influence tumor hypoxia [31], we next measured hypoxia in these tumors by the relative binding of the marker drug pimonidozole. We found that the MRPL28 knockdown tumors had a significant reduction in the overall hypoxic fraction within the tumor when compared to sizxe matched controls (figure 5B). Lastly, we examined the tumors to determine if the reduced hypoxia had a significant effect on tumor growth parameters. While we found no difference in the fraction of tumor cells that were apoptotic (data not shown), we did find an increase in the fraction of cells that were proliferating as measured by uptake of the radiotracer 18F fluorothymidine (Figure 5C left panel and figure S4). To confirm that the 18F thymidine uptake represented increased proliferation, we also measured proliferation by staining histologic sections with anti-Ki67 antibody, and determined a significant increase in the knockdown tumors (figure 5C right panel).

**Figure 6:**
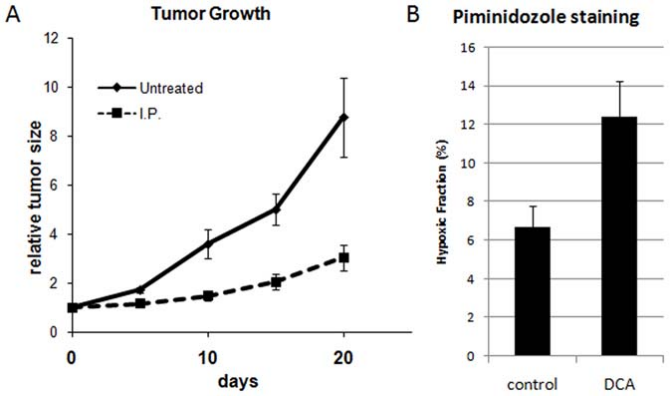
Pharmacologic increase in oxygen consumption results in slower tumor growth. Figure 6A) SU86 tumors were grown in nude mice and when tumors reached 100–200 mm3, animals were divided into control (PBS) and treated (50 mg/kg DCA IP) groups. Animals were treated daily from day 2 to 16 and tumor volumes measured as indicated. Figure 6B) At day 21 animals were injected with DCA, one hour later by Pimonidozole and 3 hours later sacrifieced. Tumors were explanted and a single cell suspension generated that was stained with FITC-labelled anti-pimonidozole antibody. Cells were analyzed by FACS and the fraction staining >200 (AUs) is indicated.

We next asked if we could phenocopy this genetic characteristic of increased mitochondrial function and decreased tumor growth pharmacologically. One drug that has been shown to increase mitochondrial activity is dichloroacetate (DCA). DCA is a pyruvate-mimetic that can increase pyruvate dehyrogenase activity in hypoxic cells by inhibiting pyruvate dehydrogenase kinases [31]. The increase in PDH activity increases oxygen consumption, and hypoxia, in the hypoxic tumor. Although additional activities of DCA have been reported [32], [33], we treated animals bearing SU86 tumors with either PBS or 50 mg/kg DCA on a daily schedule. The tumors in the DCA treated group showed significantly slower growth than the control tumors (figure 6A). At the end of the experiment the animals were injected with pimonidozole and the tumors harvested and stained for pimonidozole uptake. Figure 6B shows that even though the control tumors were much larger, there was significantly more hypoxia in the DCA treated tumors (Figure 6B).

## Discussion

There are numerous reports of mutations in the mitochondrial genomes of tumor cells, or in enzymes of the mitochondrial TCA cycle, suggesting that these alterations are beneficial for tumor growth. However, these mutations can accumulate with age, and so it is difficult to show that they have a functional role during tumorigenesis. Additionally, it is not clear if these mutations have additional, non-metabolic effects, such as altering apoptotic sensitivity [34], or interfering with the HIF1 signaling pathway [13]. Conversely, it is difficult to reduce mitochondrial function without increasing glycolysis as these are the only two mechanisms for generating ATP in the cell. This work supports the hypothesis that it is the mitochondrial activity that is first reduced that causes the increase in glycolytic activity secondarily. Furthermore, it provides a functional link between the altered tumor metabolism described by Warburg and the increased tumor growth in vivo.

Tumor cell metabolism is designed to support the synthesis of a complete daughter cell each cell cycle. This requires the coordinated production of the biochemical precursors necessary for macromolecular synthesis. The metabolic pathways that generate energy intersect most directly with the pathways responsible for nucleic acid synthesis (the use of glucose-6-phosphate in the pentose shunt) and lipid synthesis (acetylCoA from the mitochondria for 2 carbon lipid extension at fatty acid synthase) [35]. Shifting substrates from energy production to molecular synthesis could provide tumor cells with additional capacity for macromolecular production. Activation of the AKT oncogene has been shown to alter metabolism and shift the flow of precursors into molecular synthesis[36]. However, this does not appear to be the case for the mutations that we report here because they actually slow the growth of tumor cells in vitro in normoxia where there is excess glucose and oxygen in the media [37]. It is only in the hypoxic environment where oxygen is limiting that the mutations are beneficial to cellular proliferation (figure 4).

The HIF1 transcription factor has also been shown to influence the metabolic choices within the tumor cell. This adaptive response slows oxygen consumption and increases glucose metabolism during hypoxia [23]. This observation is consistent with the idea that oxygen can be rate limiting for growth in vivo, and slowing its consumption contributes to accelerate tumor proliferation. The HIF1 data has also been interpreted by some to support a model in which reduced mitochondrial function leads to reduced generation of toxic oxygen byproducts, or reactive oxygen species (ROS), and therefore decreases cellular death [10], [12]. It is true that slowing the oxygen flux through the mitochondria will slow the metabolic production of ROS, but the role of ROS in killing hypoxic cells is controversial [38], [39], [40]. Our data suggests that ROS are not toxic to the cells in either normoxia or hypoxia. The addition of the radical scavenger N-acetyl cysteine (NAC) to the media does not protect the mutant cells during growth at 21% oxygen (figure 4). Additionally, there is not increased death of cells in the wild type tumors as measured by staining with TUNEL for apoptosis (data not shown).

The hypothesis that oxygen can be limiting to the growth of human tumors has been presented. What has been difficult to generate is a genetic model system that can test this independent of other activities. In the 1950's Thomlinson and Gray made the observation that human tumor cells grew in vivo around blood vessels but only for a limited distance [41]. This architecture of the “tumor cord” has been shown to be a function of the need for tumor cells to extract oxygen from the blood vessel. However, oxygen is consumed as it diffuses through the cells. The diffusion distance of approximately 100–200 microns is a function of the rate of delivery from the blood vessel and the rate of consumption within the cell [42], [43]. This oxygen diffusion limit limits the proliferation of the tumor cells. However, if the tumor cells are able to decrease the rate with which they consume oxygen, the diffusion limit will be increased [44]. Active downregulation of mitochondrial function by HIF1 can accomplish this reduction in oxygen consumption similarly to the mutations that we generated. Pharmacologically blocking this adaptive response with DCA leads to increased oxygen consumption and reduced tumor growth. The pancreatic tumor cells seem to be sensitive to this intervention, and this cell-type sensitivity should be taken into account as clinical trials progress with this drug [45].

Oxygen is required for non-mitochondrial activities that are required for growth of tumor cells. This requirement for oxygen allows tumor cells to grow in hypoxia, but cannot support growth in anoxia. This anoxic death can be observed in vivo as well as in vitro [27], [46]. As it has been shown in yeast, loss of mitochondrial function allows slow growth of “petite” colonies on a source of glucose, but will not support growth in anoxia [47]. Several processes have been identified that require molecular oxygen as a substrate, such as sterol synthesis, or oxidative protein folding [48], [49]. Neither of these processes can occur in the total absence of oxygen, but can efficiently proceed in moderate hypoxia. Furthermore oxidases, hydroxylases, and histone demethylases all require molecular oxygen as a substrate [44]. These oxygen-dependent cellular functions are necessary for the growth of cells within the tumor. This model of how decreased mitochondrial function provides tumor cells with additional substrates (ie oxygen) for macromolecular synthesis could be considered an outgrowth of the model in which increased glycolysis provides tumor cells with increased small molecule intermediates for increased macromolecular synthesis [35].

## Materials and Methods

### Cell lines and cell culture

Su86.86 and MiapaCa2 human pancreatic cancer cells were obtained from the American Type Culture Collection (ATCC, Manassas, VA). Su86.86 were grown in RPMI1640 supplemented with sodium pyruvate and Hepes and 10% fetal bovine serum (FBS), while MiapaCa2 cells were grown in Dulbecco's Modified Eagle's Media (DMEM) supplemented with 10% FBS. For moderate hypoxia, cell culture dishes were placed into an Invivo_2_ humidified hypoxia workstation (Ruskinn Technologies, Bridgend, UK) at the indicated oxygen concentrations.

### Plasmids and siRNAs

Full-length human UCP1 cDNA was obtained from the mammalian gene collection (MGC), through the ATCC, and was cloned into pBabePuro vector. For knockdown experiments, the target sequences used were: Complex IV subunit IV, CACTGAAGGAGAAGGAGAA, MRPL12, CAACGAGCTCCTGAAGAAA. MRPL28 shRNA1, TACAAGGAATTTGCCATCCCAGAGGA, and shRNA2, GCCAAGTTCAAGATCAAC. For construction of stable knockdown cell lines, the sequences listed above were cloned into pSIREN-RetroQ vector. All the knock down cells were selected with 1 µg/ml puromycin for at least one week and a pool of the infected cells were used for each of the experiments.

### Oxygen consumption measurements and Pimonidozole staining

Cells were trypsinized and suspended at 6×10^6^ cells per ml in DMEM + 10% FBS. Oxygen consumption was measured in a 0.5 ml volume using an Oxytherm electrode unit (Hansatech, Norfolk, UK). This system employs a Clark-type oxygen electrode to monitor the dissolved oxygen concentration in a sealed measurement chamber over time. The data are exported to a computerized chart recorder (Oxygraph 1.01, Hansatech, Norfolk, UK), which calculates the rate of oxygen consumption. A small stir bar maintains the cells in suspension, and a peltier heating block maintains the temperature at 37°C. Since the electrode consumes oxygen during measurement, the rate of oxygen drop in 0.5 ml of DMEM media without cells was established and subtracted from the total oxygen consumption rates for the cell suspensions. Pimonidozole staining was performed on animals injected with 60 mg/kg pimonidozole 3 hours prior to sacrifice. Pimonidozole was detected by immunofluorescence of frozen sections (figure 5) or by flow cytometry of a single cell suspension (figure 6). Cells were blocked with PBS containing 0.1% triton and 3% fat free milk, and detected with FITC-labeled Hypoxyprobe-1 antibody. Detection threshold was determined by staining cells from animals that did not receive drug.

### siRNA library screen

The GeneNet™ Human Druggable Genome siRNA Library contains 43,800 siRNA templates targeted to 8,500 well-characterized human genes (System Biosciences, CA). For most of the target genes, there are five different siRNA sequences. The library is cloned into the FIV-based pSIF1-H1-Puro shRNA Expression Vector, which confers resistance to the antibiotic puromycin. After transducing the cells with pre-packaged VSV-G pseudotyped virus containing the siRNA library, the MiaPaCa2 cells were selected with puromycine for 5 days. Three million cells with puromycine resistance were injected into Scid mice. One and four weeks later, the mice were sacrificed and RNA from tumors was extracted. The lentiviral inserts—containing the siRNA templates were recovered by PCR and identified using the Human Genome Focus GeneChip® Array (Affymetrix, CA).

### microPET Imaging and Image Analysis

microPET scans were performed on a microPET R4 rodent model scanner (Concorde Microsystems Inc.). Mice bearing tumors on the shoulders were injected through tail-vein of 200 µCi of radiotracer (^18^F-FDG) under isoflurane anesthesia. The microPET data acquisition was started 60 min after injection. The images were reconstructed by a 2-dimensional ordered-subsets expectation maximum (OSEM) algorithm and no correction was necessary for attenuation or scatter.

### Western blotting

In brief, treated cells were harvested directly in buffer containing 8 M urea and 15 mM b-mecaptoethanl, protein concentrations were quantitated, 50–100 µg were electrophoresed on a reducing Tris-Tricine gel, and electroblotted to PVDF membrane. Antibodies used were MRPL12 (1∶200, Abnova), MRPL28 (1∶200, Genway), HSP60, ATPaseIP, COXIII and Complex IV subunit IV (1∶500, Mitosciences). Primary antibodies were detected with species-specific secondary antibodies labeled with peroxidase (Vector labs 1∶3000) and visualized with Supersignal substrate (Pierce) with films.

### Immunocytochemistry and fluorescence microscopy

Cells were plated on glass multiwell chamber slides (Nalge Nunc, Naperville, IL) in DMEM + 10% FBS. For cytochrome c immunocytochemistry, chamber slides were fixed in 4% paraformaldehyde, blocked in PBS Tween milk (0.2% Tween 20, 5% nonfat dry milk, in PBS) for 1 h. Cytochrome c was visualized using a mouse monoclonal antibody 1∶250 (BD Pharmingen, San Diego, CA) and an anti-mouse Alexa 488 secondary antibody 1∶500 (Molecular Probes).

### Metabolite Assays

2-Deoxyglucose uptake assay: 2-[1,2,-3H (N)]-Deoxy-D-glucose was purchased from PerkinElmer. Cells were plated in six-well plates and 24 hours later were washed with PBS and fresh media containing 0.5 mM glucose and 0.5 µCi ^3^H-2DG were added for 30 min. At the end of the incubation the monolayers were rinsed three times with cold PBS, lysed with 0.2 M NaOH/0.1% SDS and radioactivity measured with a liquid scintillation counter. Protein concentration was measured in parallel series of samples. Lactate measurement assay: Lactate concentrations in the culture media were measured using a colorimetric assay kit (SUNY at Buffalo).

### Data analysis

Oxygen consumption experiments were repeated three times in duplicate, survival was measured three times in triplicate, reporter assays were repeated two times in quadruplicate. Tumor growth was determined in two separate experiments with 5 tumors per group. Error bars represent the SEM.

## Supporting Information

Figure S1 Table describing the targeting sequences of the ShRNA hairpins that scored the most consistent enrichment when comparing 1 and 4 weeks in vivo to the same time in vitro.
(0.79 MB TIF)

Figure S2 Growth characteristics of the truncated MRPL28-FLAG expressing Su86 cells grown in vivo (left panel). Oxygen consumption data from the same overexpressing cells (Right panel)
(0.26 MB TIF)

Figure S3 Analysis of the MRPL12 knockdown tumors ex vivo. Tumors were explanted and a portion used to determine knockdown efficiency by Western Blot (left panel) and a portion used to measure oxygen consumption (tight panel). Minced tumor sections were weighed and placed in the oxygen electrode to determine consumption per mg of tumor.
(0.31 MB TIF)

Figure S4 Quantitation of the mean FLT signal from the Miapaca2 control tumors and MRPL28 knockdown tumors imaged in figure 5c.
(0.14 MB TIF)
