# Supplementary figures and images for: Oxygen Consumption Can Regulate the Growth of Tumors, a New Perspective on the Warburg Effect
Source: PLoS One. 2009 Sep 15;4(9):e7033. doi: 10.1371/journal.pone.0007033 (PMC2737639; doi:10.1371/journal.pone.0007033)

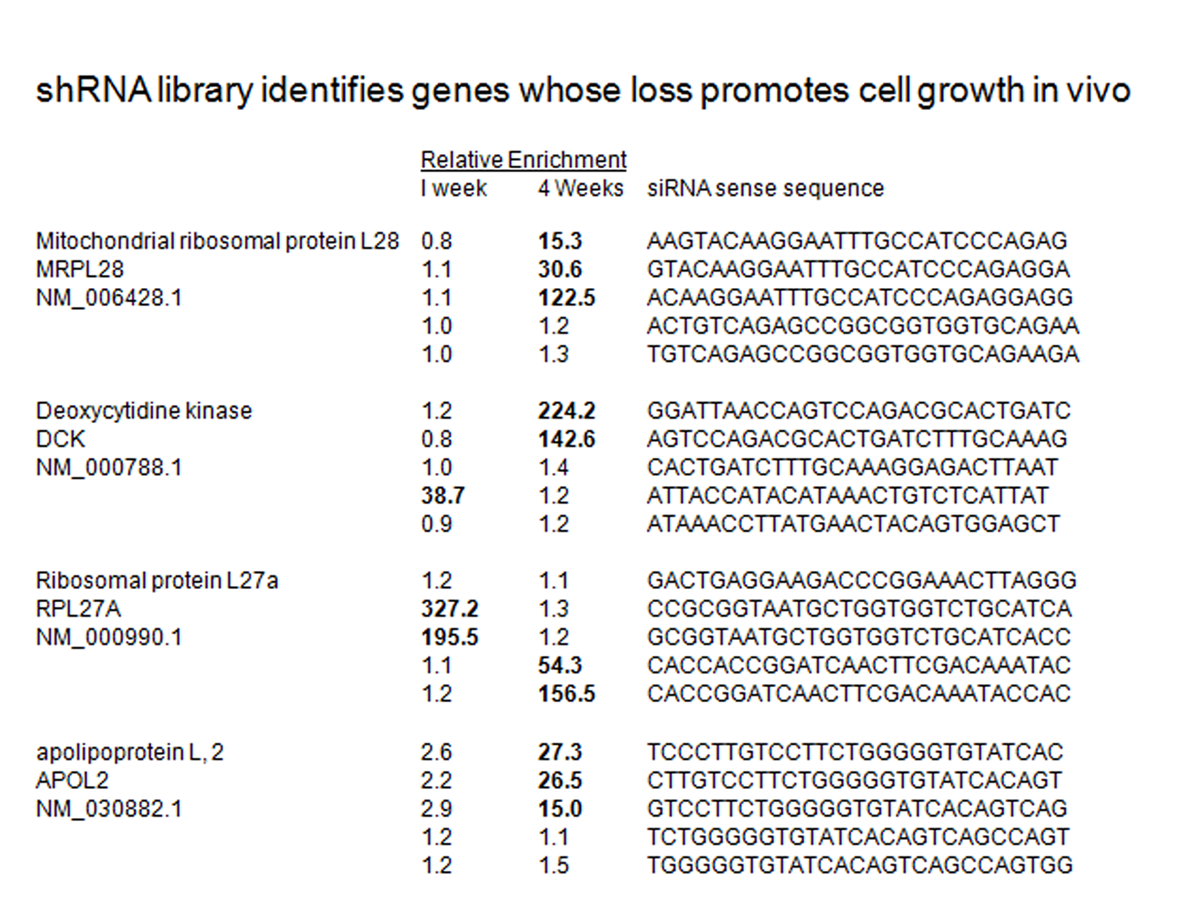

Supplement: Figure S1 — Table describing the targeting sequences of the ShRNA hairpins that scored the most consistent enrichment when comparing 1 and 4 weeks in vivo to the same time in vitro. (0.79 MB TIF) [file pone.0007033.s001.tif]

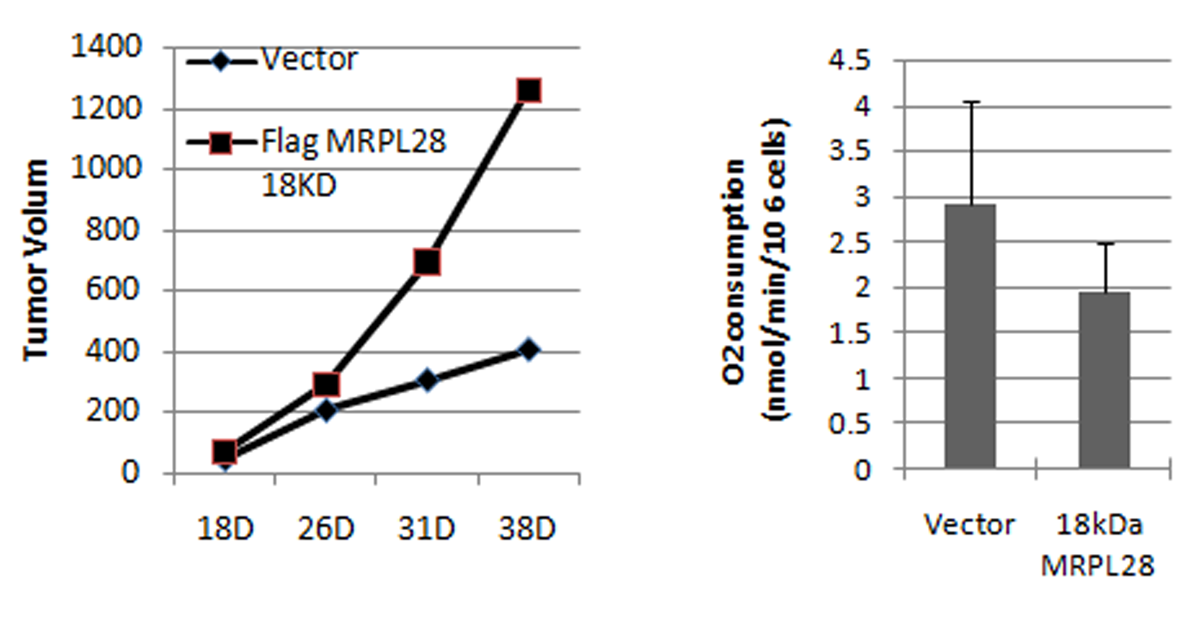

Supplement: Figure S2 — Growth characteristics of the truncated MRPL28-FLAG expressing Su86 cells grown in vivo (left panel). Oxygen consumption data from the same overexpressing cells (Right panel) (0.26 MB TIF) [file pone.0007033.s002.tif]

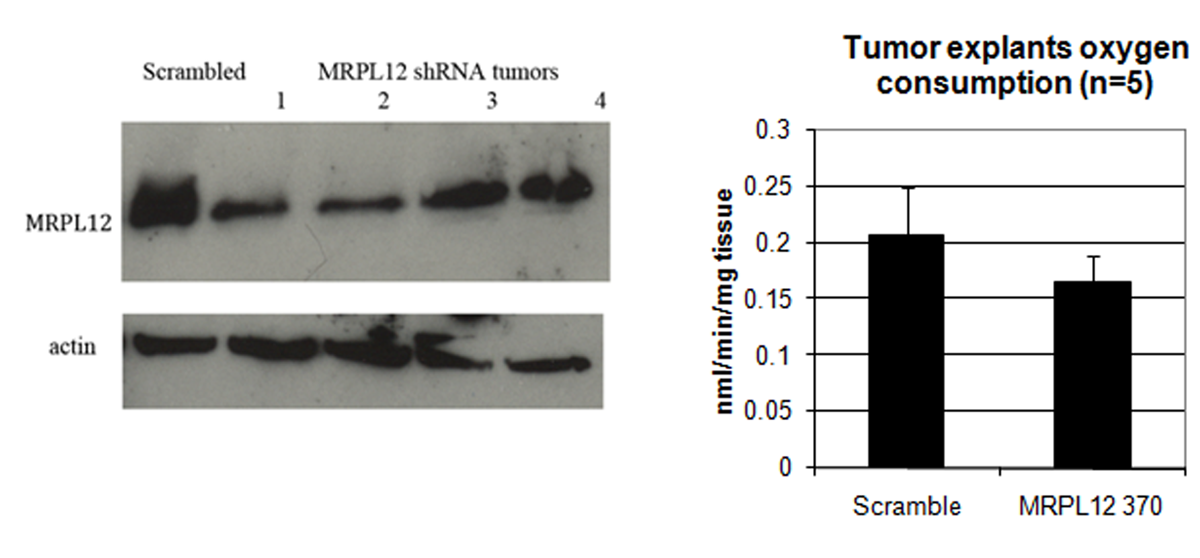

Supplement: Figure S3 — Analysis of the MRPL12 knockdown tumors ex vivo. Tumors were explanted and a portion used to determine knockdown efficiency by Western Blot (left panel) and a portion used to measure oxygen consumption (tight panel). Minced tumor sections were weighed and placed in the oxygen electrode to determine consumption per mg of tumor. (0.31 MB TIF) [file pone.0007033.s003.tif]

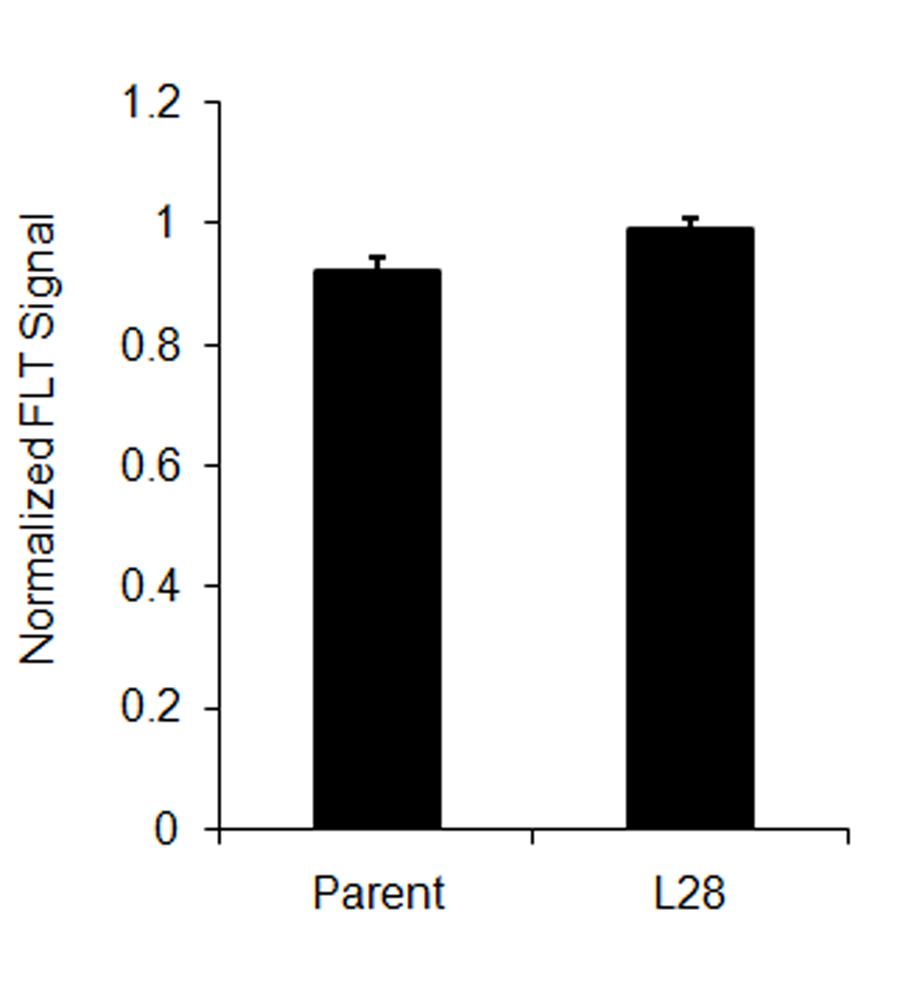

Supplement: Figure S4 — Quantitation of the mean FLT signal from the Miapaca2 control tumors and MRPL28 knockdown tumors imaged in figure 5c. (0.14 MB TIF) [file pone.0007033.s004.tif]
